# Sildenafil-Induced Revascularization of Rat Hindlimb Involves Arteriogenesis through PI3K/AKT and eNOS Activation

**DOI:** 10.3390/ijms23105542

**Published:** 2022-05-16

**Authors:** Celine Baron-Menguy, Arnaud Bocquet, Alexis Richard, Anne-Laure Guihot, Bertrand Toutain, Pierre Pacaud, Celine Fassot, Gervaise Loirand, Daniel Henrion, Laurent Loufrani

**Affiliations:** 1 MITOVASC, Equipe CarMe, CNRS 6015, Inserm 1083, Angers Université, 49100 Angers, France; celine.menguy@univ-nantes.fr (C.B.-M.); arnaud.bocquet@gmail.com (A.B.); alexis.richard@univ-angers.fr (A.R.); anne-laure.guihot@univ-angers.fr (A.-L.G.); bertrand.toutain@univ-angers.fr (B.T.); celine.fassot@inserm.fr (C.F.); daniel.henrion@univ-angers.fr (D.H.); 2 Nantes Université, CHU Nantes, CNRS, INSERM, L’Institut du Thorax, F-44000 Nantes, France; pierre.pacaud@univ-nantes.fr; 3 CHU d’Angers, 49100 Angers, France; gervaise.loirand@univ-nantes.fr

**Keywords:** remodeling, vasodilation, VEGF, sildenafil, neovascularization

## Abstract

Hypoxia and inflammation play a major role in revascularization following ischemia. Sildenafil inhibits phosphodiesterase-5, increases intracellular cGMP and induces revascularization through a pathway which remains incompletely understood. Thus, we investigated the effect of sildenafil on post-ischemic revascularization. The left femoral artery was ligated in control and sildenafil-treated (25 mg/kg per day) rats. Vascular density was evaluated and expressed as the left/right leg (L/R) ratio. In control rats, L/R ratio was 33 ± 2% and 54 ± 9%, at 7- and 21-days post-ligation, respectively, and was significantly increased in sildenafil-treated rats to 47 ± 4% and 128 ± 11%, respectively. A neutralizing anti-VEGF antibody significantly decreased vascular density (by 0.48-fold) in control without effect in sildenafil-treated animals. Blood flow and arteriolar density followed the same pattern. In the ischemic leg, HIF-1α and VEGF expression levels increased in control, but not in sildenafil–treated rats, suggesting that sildenafil did not induce angiogenesis. PI3-kinase, Akt and eNOS increased after 7 days, with down-regulation after 21 days. Sildenafil induced outward remodeling or arteriogenesis in mesenteric resistance arteries in association with eNOS protein activation. We conclude that sildenafil treatment increased tissue blood flow and arteriogenesis independently of VEGF, but in association with PI3-kinase, Akt and eNOS activation.

## 1. Introduction

Shear stress generated by blood flow is the major stimulus for the basal production of NO by the endothelium [1] and for the control of eNOS expression [2]. Thus, flow might play a central role in both angiogenesis and arteriogenesis following ischemia. A chronic increase in blood flow induces a rise in arterial diameter (outward hypertrophic remodeling) in order to normalize shear stress [2]. This remodeling, also called arteriogenesis, requires the production of NO (via eNOS activation) as well as Akt and phosphatidylinositol 3-kinase (PI3K) activation [2,3]. NO produced by flow-stimulated endothelial cells induces cGMP production; this activates many cellular processes such as cell proliferation [4]. Furthermore, macrophage is one of the important players in immune response which secretes many important factors including cytokines and growth factors to regulate angiogenesis and arteriogenesis, acting directly or indirectly on the vascular cells [5].

Despite advances in the interventional and medical treatment of peripheral arterial disease, chronic tissue ischemia remains an unmet medical need in which tissue revascularization plays an essential role. Indeed, leaking of revascularization involves severe and irreversible damages. Hypoxia and inflammation play a major role in the revascularization process following ischemia [6]. In ischemia-induced revascularization, angiogenesis and arteriogenesis are both involved, although they cannot be easily distinguished in vivo.

Angiogenesis refers to the growth of new blood vessels from pre-existing ones. In response to an angiogenic stimulus, endothelial cells invade the surrounding extracellular matrix, migrate to the site of recruitment, proliferate and finally stabilize the newly formed vessel [7]. Hypoxia induces angiogenesis mainly through an increase in vascular endothelial growth factor (VEGF), which sequentially activates Akt, endothelial nitric oxide synthase (eNOS) and guanosine 3′,5′-cyclic monophosphate (cGMP)-dependent protein kinase (PKG) [8,9,10,11]. Recently, Falero-Diaz et al. showed that ischemia induced monocytes reprogramming so that these could promote arteriogenesis at distant locations and during subsequent ischemic events [12].

In smooth muscle cells, cGMP is rapidly catabolized by phosphodiesterase 5A (PDE5A) [13]. Recent studies have demonstrated that elevated cGMP levels in cerebral tissues may be involved in angiogenesis following embolic stroke in the rat [14]. Sildenafil is a PDE5A inhibitor highly specific for cGMP hydrolysis [15] and its administration increases intracellular cGMP concentration [14]. It has been successfully used in patients suffering from pulmonary arterial hypertension [16,17] and erectile dysfunction with cardiovascular risk factors [18]. Moreover, El-Naa et al. shed light on the antitumor activity of sildenafil and its possible use to amplify the antitumor effect of conventional chemotherapeutic agents. These effects might be related to the antiangiogenic, antiproliferative, and pro-apoptotic activities of sildenafil [19]. Sildenafil has also shown promising results in the management of cardiac ischemia-reperfusion injury and perivascular arterial disease. Indeed, sildenafil increases ischemia-induced revascularization and blood flow in the ischemic hindlimb in the mouse. This effect of sildenafil depends on PKG activation but is independent to NO [20]. Nevertheless, NO enhances angiogenesis via the production of VEGF and cGMP in rats suffering from stroke and treated with sildenafil [21]. Furthermore, donepezil (a specific and reversible inhibitor of acetylcholinesterase) treatment influences, to a greater extent, angiogenesis, causing a significant increase in capillary density, suggesting that cholinergic stimulation may be involved in the process [22]. Consequently, the mechanisms by which sildenafil confers protection during ischemic diseases requires further investigation, especially when it is used chronically. Recently, it has been demonstrated that sildenafil, as a potent stimulator of angiogenesis, accelerates and better repairs wound healing through the regulation of proinflammatory cytokines and collagen/TGF-β1 pathways [23].

The aim of the present study was to determine the mechanism involved in the protective effect of in vivo treatment of sildenafil in peripheral ischemia induced by the ligation of the left femoral artery in rats.

## 2. Results

### 2.1. Body Weight and Blood Pressure of Control and Sidenafil Treated-Rats

Rat body weight was not affected by sildenafil after 7 days (272 ± 6 g vs. 279 ± 8 g in control group, *n* = 10) or 21 days (418 ± 5 g vs. 426 ± 5 g in control group, *n* = 10). Sildenafil did not significantly affect mean arterial blood pressure after 7 days (107 ± 2 mmHg vs. 107 ± 3 mmHg in control group, *n* = 10) but significantly decreased it after 21 days of treatment (100 ± 2 mmHg vs. 115 ± 4 mmHg in control group, *p* < 0.05, *n* = 10).

### 2.2. Plasma and Aortic cGMP Levels

As expected, rats treated with sildenafil had a higher cGMP concentration in the aorta (63.96 ± 10.18 nmol/mg vs. 20.48 ± 0.88 nmol/mg in control group, *p* < 0.05, *n* = 5) and in the plasma (32.82 ± 4.69 nmol/mL vs. 17.12 ± 2.48 nmol/mL in control group, *p* < 0.05, *n* = 5) compared to control rats (Table 1 and Table 2).

### 2.3. In Vivo Treatment of Sildenafil Enhanced Post-Ischemic Revascularization

**Figure 1:**
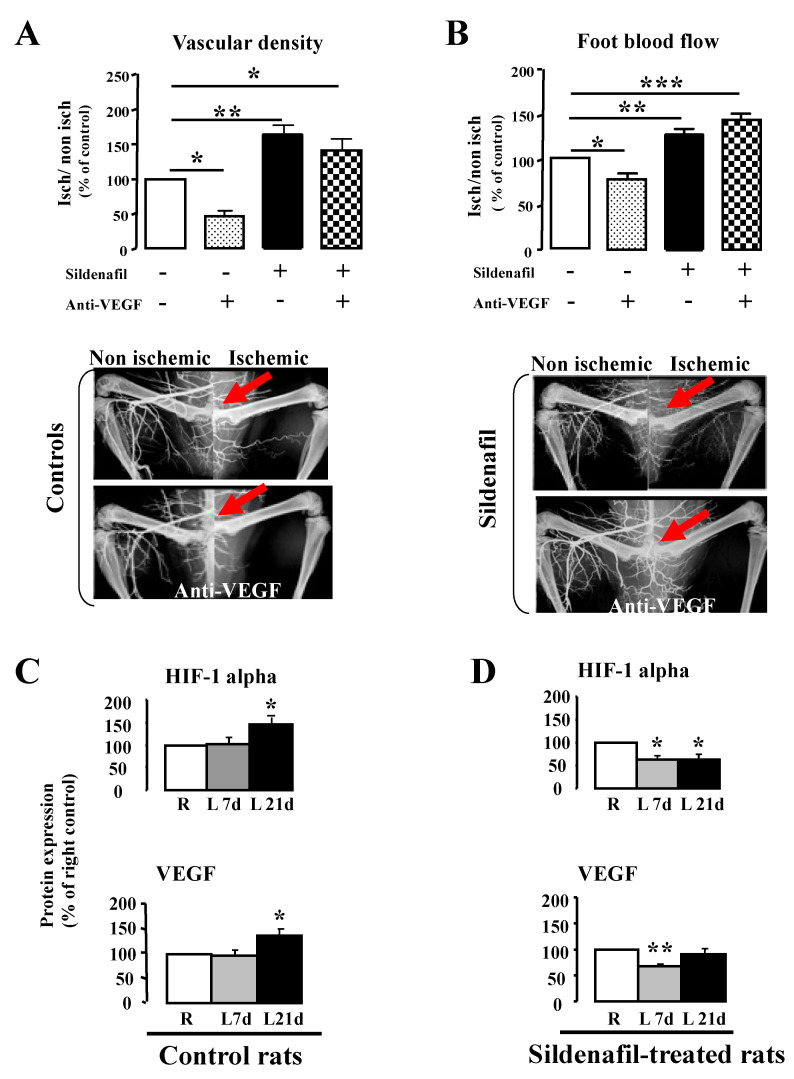
Evaluation of revascularization 7 days after femoral artery ligation (red arrows) in control and sildenafil-treated rats in the presence or absence of VEGF neutralizing treatment. Vascular density (*n* = 6) and blood flow (*n* = 6) are shown with typical X-rays in panels (**A**,**B**), respectively. (**C**,**D**): Protein expression ratio of HIF1α and VEGF in the ischemic hindlimb compared to right non-ischemic hindlimb in control (**C**) and sildenafil-treated rats (**D**). Mean ± SEM are presented (*n* = 10 per group). Values are expressed as the ischemic/non-ischemic leg ratio (% of controls). * *p* < 0.05, ** *p* < 0.01, *** *p* < 0.001 versus control group.

We first evaluated revascularization 7 days after the left femoral artery ligation. Vascular density in the control group was lower in the ischemic (left, L) leg compared to the non-ischemic (right, R) leg with a L/R ratio of 33 ± 2%. Sildenafil had a beneficial effect on revascularization after 7 days of treatment, significantly increasing the L/R ratio to 47 ± 4% (*p* < 0.01), corresponding to a 1.5-fold increase (Figure 1A). To assess the involvement of VEGF in this process, both control and sildenafil-treated rats were simultaneously treated with a VEGF neutralizing antibody. In the control group, the antibody significantly decreased vascular density (0.48-fold, *p* < 0.05), whereas in sildenafil-treated rats, the antibody had no significant effect on revascularization (Figure 1A).

**Figure 2:**
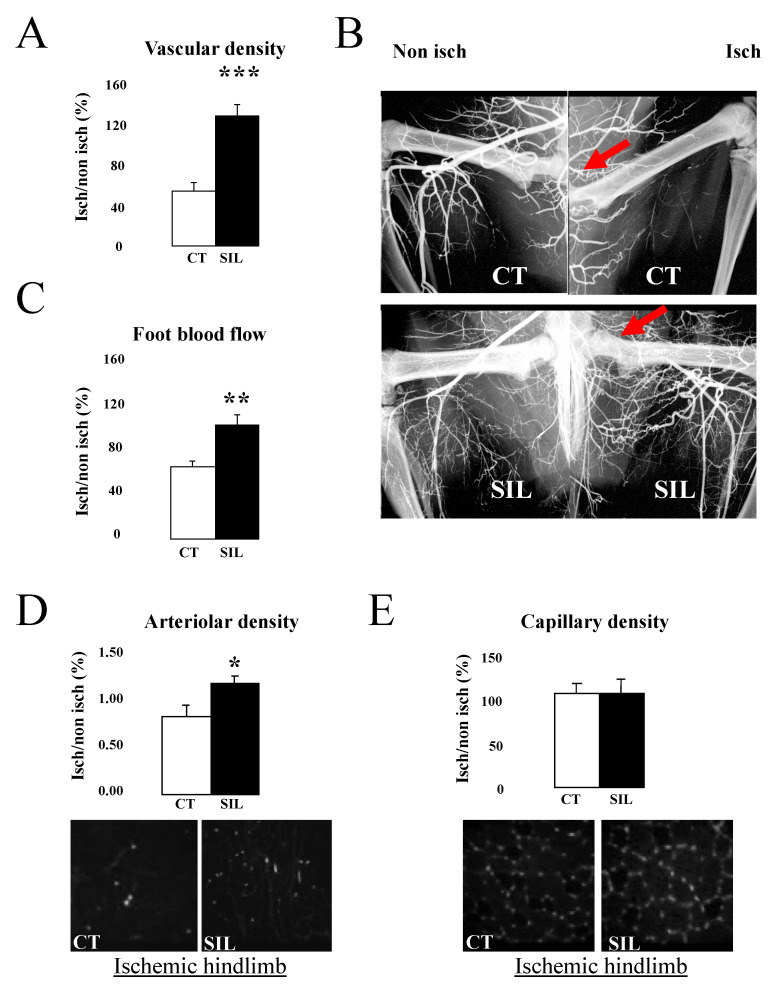
Quantitative evaluation of leg neovascularization 21 days after femoral artery ligation (red arrows) in sildenafil-treated and control rats. Vascular density (*n* = 12), typical radiograms (*n* = 12), blood flow (*n* = 6), arteriolar (*n* = 6) and capillary densities (*n* = 6) are shown in panels (**A**–**E**). Ischemic/non-ischemic leg ratio (%) values are expressed in mean ± SEM. * *p* < 0.05, ** *p* < 0.01, *** *p* < 0.001 versus control group. CT: Control SIL: Sildenafil.

After 21 days of ligation (Figure 2), in the control group, vascular density in the ischemic leg was lower than in the non-ischemic leg, with a L/R ratio of 54 ± 9%. Chronic sildenafil treatment increased by 2.40-fold the L/R ratio (128 ± 11%, compared to control, *p* < 0.01, *n* = 6). The effect of sildenafil on post-ischemic revascularization was confirmed by hindlimb perfusion measurements and by evidence of increased arteriolar density. Capillary density was the same as in the control group.

Compared to the non-ischemic leg, blood flow in the ischemic leg was significantly reduced in the control group (L/R ratio: 36 ± 3%). After VEGF neutralization, the L/R ratio was significantly decreased (0.77-fold, *p* < 0.05). Chronic treatment with sildenafil significantly increased the leg blood flow (+53 ± 5% compared to untreated rats, *p* < 0.01). However, blood flow was not affected by the VEGF neutralizing antibody, suggesting a VEGF-independent effect of sildenafil (Figure 1B).

In order to confirm the data obtained using microangiography, we examined arteriolar and capillary densities in both legs. After 7 days ligation, the arteriolar density (L/R ratio) was significantly higher in the sildenafil group than in controls (128.3 ± 5.5% vs. 103.1 ± 6.9%, respectively, *p* < 0.05). In the control group, the neutralizing VEGF treatment significantly reduced the arteriolar density (0.67-fold), whereas no significant effect was observed after sildenafil treatment (arteriolar density increased by 1.26-fold). Moreover, capillary density was not affected by sildenafil (136.9 ± 18.1% vs. 172.0 ± 35.6% in the control group). In the control group, the VEGF neutralizing treatment significantly reduced the capillary density (65.8 ± 12.4%, *p* < 0.05, *n* = 5), whereas in sildenafil group, no effect was observed (125.6 ± 4.8%, *n* = 6). We next performed a biochemical study in order to assess the involvement of HIF-1α and VEGF in revascularization with or without sildenafil. In the control group, both HIF-1α and VEGF expression increased in the ischemic leg after 21 days of ligation. In sildenafil-treated rats, HIF-1α and VEGF expression were significantly decreased after 7 days (*n* = 10) (Figure 1C,D).

### 2.4. Analysis of the NO Pathway Using Western Blot Analysis

**Figure 3:**
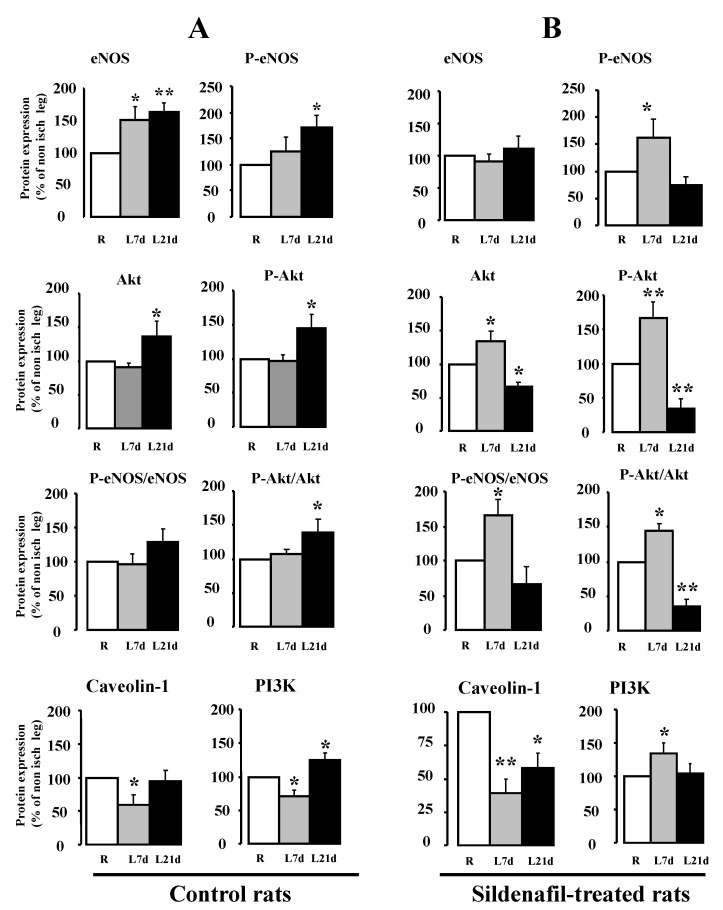
Evaluation of revascularization for eNOS (*n* = 12), Phospho-eNOS (*n* = 12), caveolin-1 (*n* = 8), Akt (*n* = 9), Phospho-Akt (*n* = 9), and PI3 kinase (*n* = 9) in the ischemic (left or L) leg compared to the non-ischemic (right or R) leg in control (**A**) and sildenafil-treated rats (**B**), at 7 (7d) or 21 (21d) days after ligation. Protein expression ratio are expressed as percentage of the right leg measurement. Mean ± SEM are presented. * *p* < 0.05, ** *p* < 0.01 versus non-ischemic right leg.

Protein expression level (Figure 3) was determined in the ischemic (left) and non-ischemic (right) legs and expressed as a percentage of the non-ischemic leg. In the control group, the activities of eNOS and Akt were not significantly affected by ligation after 7 days, although the expression of PI3K was significantly decreased (*p* < 0.05). After 21 days, ligation induced a significant rise in the expression levels of eNOS (1.6-fold, *p* < 0.01), P-eNOS (1.7-fold, *p* < 0.05) Akt (1.4-fold, *p* < 0.05), P-Akt (1.4-fold, *p* < 0.05) and PI3K (1.3-fold, *p* < 0.05) in the ischemic leg, compared to the non-ischemic leg (Figure 3, left panel).

In sildenafil-treated rats, eNOS protein expression was not modified 7 and 21 days after ligation; however, P-eNOS expression was significantly increased in the ischemic leg (1.7-fold, *p* < 0.05) in association with a decreased caveolin-1 expression level (0.5-fold, *p* < 0.01) at day Chronic sildenafil also induced a significant increase in Akt, P-Akt and PI3K expression level (1.3-, 1.7- and 1.8-fold, respectively) in the ischemic hindlimb at day After 21 days of treatment with sildenafil, the expression level of eNOS, P-eNOS, Akt, P-Akt and caveolin-1 was strongly reduced in the ischemic leg, suggesting a down-regulation of the pathway.

### 2.5. Endothelial Cell Migration

**Figure 4:**
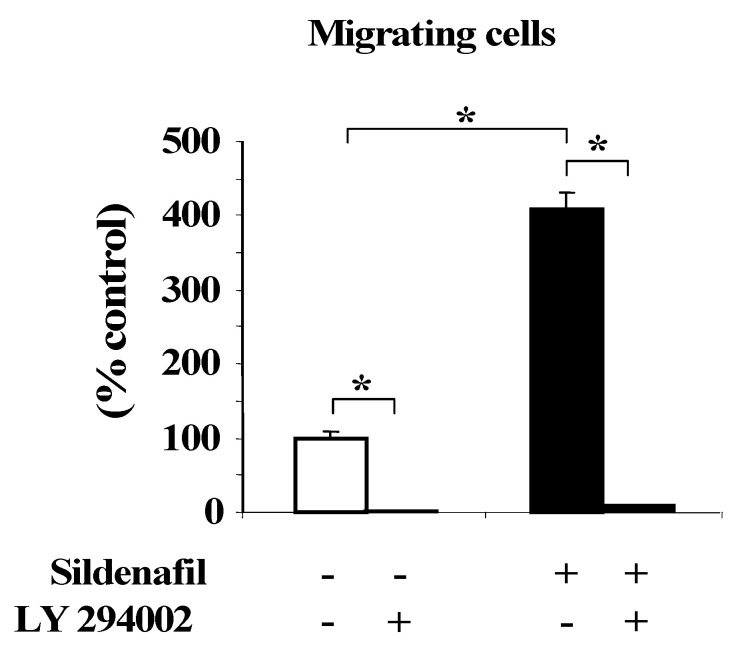
Evaluation of endothelial cell migration using a Boyden chamber assay in control and sildenafil-treated (10 µM, for 8 h) cells in the presence or absence of the PI3 kinase inhibitor LY294002 (25 µM). Results are expressed as a percentage of control group (* *p* < 0.05).

Sildenafil (10 µM) [24] strongly stimulated endothelial cell migration (compared to untreated cells, *p* < 0.05). When cells were pretreated with the PI3K inhibitor LY294002 (25 µM), this cell migration did not occur (Figure 4).

### 2.6. In Vico Effect of Sildenafil on the Non-Ischemic Hindlimb

**Figure 5:**
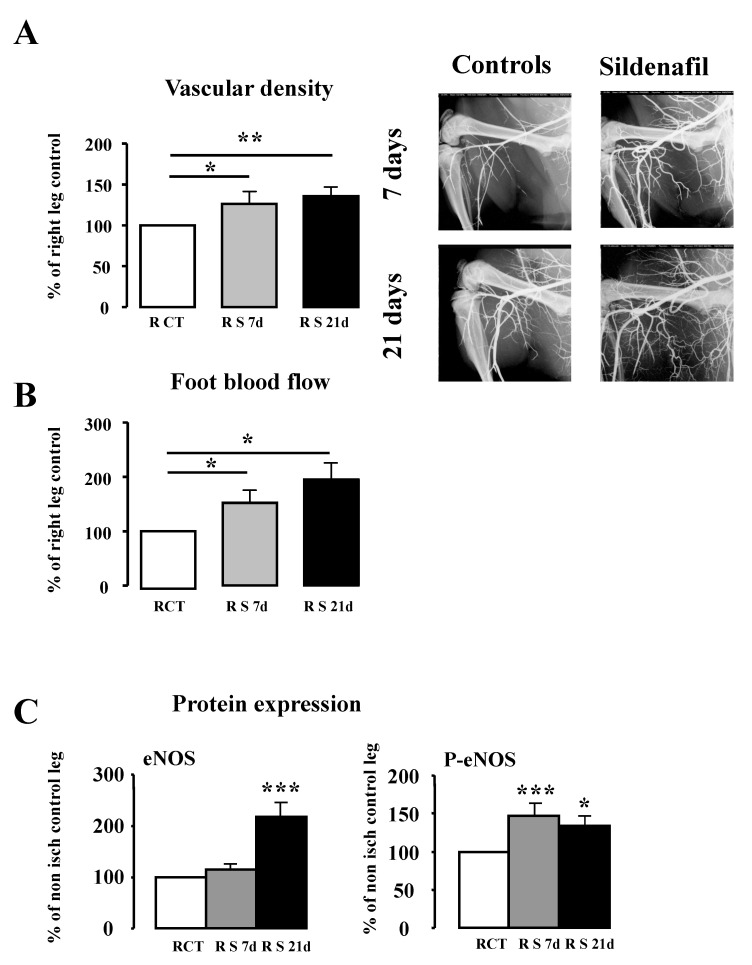
Evaluation of revascularization after 7 and 21 days of sildenafil treatment on vascular density (**A**), blood flow (**B**) and eNOS and P-eNOS protein expression ratio (**C**) in the non-ischemic (right) leg. Protein expression ratio are expressed in mean ± SEM (*n* = 6). R: Right CT: Control S: Sildenafil d: day, * *p* < 0.05, ** *p* < 0.01, *** *p* < 0.001 versus control group.

We next studied the effect of sildenafil treatment on the non-ischemic hindlimb: vascular density was significantly increased after 7 and 21 days of treatment (1.26- and 1.36-fold, respectively, *p* < 0.05) (Figure 5A). Microangiographic measurements were associated with a significant increase in foot blood flow (*p* < 0.05) (Figure 5B). Furthermore, after 7 days of treatment, despite no difference in eNOS expression, the protein level of P-eNOS was significantly increased (1.5-fold) compared to the control group. After 21 days, sildenafil treatment enhanced both eNOS and P-eNOS protein level; this result is in accordance with the increased foot blood flow (Figure 5C).

### 2.7. In Vivo Effect of Sildenafil Treatment on Mesenteric Resistance Arteries (MRA)

**Figure 6:**
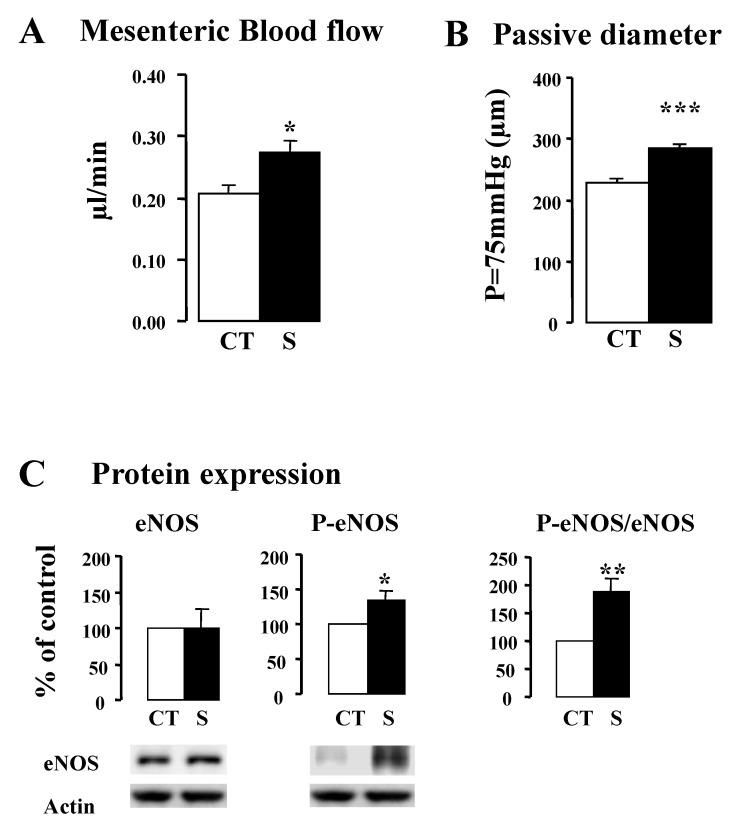
Blood flow (**A**), passive diameter (**B**), and eNOS and P-eNOS protein expression level (**C**) in MRA, in sildenafil-treated (21 days) and control rats. Values are expressed as mean ± SEM (*n* = 6 per group). CT: Control S: Sildenafil. * *p* < 0.05, ** *p* < 0.01, *** *p* < 0.001 versus control group.

To confirm the non-ischemic hindlimb results, we next examined the effect of sildenafil on isolated microvasculature. After 21 days of treatment with sildenafil, we made in vivo blood flow measurements and in vitro passive diameter measurements in MRAs. Compared to the control group, sildenafil induced a significant increase in both MRA blood flow (*p* < 0.05) (Figure 6A) and passive arterial diameter (1.25-fold, *p* < 0.001) (Figure 6B), suggesting MRA remodeling. This remodeling was associated with a significant increase in P-eNOS expression level (Figure 6C).

## 3. Discussion

Our results showed that sildenafil increased post-ischemic revascularization, probably through activation of arteriogenesis. Neither HIF-1α nor VEGF were involved in this process after 7 days of anti-VEGF treatement in vivo, whereas PI3K and Akt were implicated in sildenafil-induced revascularization and endothelial cell migration. Furthermore, sildenafil, by increasing eNOS activity, induced the outward remodeling of resistance arteries. On the other hand, the involvement of VEGF could also occur later, lagging behind the controls (after the 7 days of revascularization).

Previous studies have reported that sildenafil enhances revascularization in a time-dependent manner (7 and 21 days) in ischemic hindlimbs [20] or after experimental stroke [14], although in this latter study only a single dose of sildenafil was used. These studies do not indicate a definitive mechanism for sildenafil’s effect; however, they discriminate between angiogenesis and arteriogenesis.

Revascularization occurs in response to local tissue ischemia mainly through inflammation and hypoxia-induced signalling. One mechanism by which hypoxia activates blood vessel growth is the increase in HIF-1α, leading to the production of VEGF [25]. As expected, we found that after 21 days of femoral artery ligation, VEGF expression level significantly increased in the control group. On the other hand, in sildenafil-treated rats, HIF-1α and VEGF expression was not further increased, despite much higher revascularization. In agreement, a VEGF neutralizing antibody inhibited neovascularization in the control group but not in sildenafil-treated rats.

Sildenafil is also known to increase VEGF expression in human coronary arteriolar endothelial cells [26]. Furthermore, a single administration of sildenafil 24 h after a stroke increases VEGF synthesis, [21] and acute intravenous administration of sildenafil activates VEGF and angiopoietin-1 production in the left ventricle [27]. Elrod JW et al. also showed that the reduction in cardiac infarct size mediated by sildenafil was independent of the NO/cGMP pathway [28]. In support of our finding, Kawasaki et al. (2003) have shown that eNOS gene transfer in ischemic adductor muscles does not affect VEGF production [29].

Thus, our results demonstrated that the protective effect of a treatment with sildenafil on post-ischemic revascularization is independent of VEGF. It is highly probable that chronic treatment with sildenafil shares common features with more standard vasodilator agents, such as hydralazine (a so-called non-specific vasodilator), which also activates the NO/cGMP pathway [30] through a rise in local blood flow or shear stress, thus inducing outward hypertrophic remodeling of resistance arteries [31]. The results of the present study show a similar pattern in MRAs from sildenafil-treated rats. Furthermore, in cultured endothelial cells, NO donors induce angiogenesis [32]. Other studies performed in vitro on rat gastric microvascular endothelial cells [33] or in vivo in rat stroke models [21] suggest a pro-angiogenesis effect of NO. Nevertheless, the latter study shows that an NO donor, as well as sildenafil, increase both NO and cGMP levels, leading to increased endothelial cell proliferation and to vascular diameter enlargement around ischemic lesions [21]. This observation supports our findings, suggesting that sildenafil mainly induces arteriogenesis or arteriole diameter enlargement through the activation of the eNOS-PI3K pathway.

As sildenafil-induced revascularization was associated with a rise in PI3K, Akt and eNOS expression, we tested the involvement of the PI3K-Akt pathway in sildenafil-induced migration of HMECs. In addition, as eNOS and PI3K-Akt activation have a key role in arteriolar diameter enlargement upon flow (shear stress) stimulation, we tested for flow-mediated arteriogenesis in rats receiving sildenafil. In the rat, hindlimb revascularization due to angiogenesis cannot be distinguished from that due to arteriogenesis. We therefore collected the MRAs from the same rat treated with sildenafil and observed that their diameter was increased compared to the control rats. We have previously shown that arteriogenesis occurs in MRAs in rats treated with the vasodilator hydralazine [31] or after locally increasing blood flow [34]. The increased blood flow indeed induced arteriogenesis, which was visualized as a diameter enlargement associated with a rise in eNOS expression level [2].

Our results also demonstrated that besides having an important effect on revascularization, sildenafil increased vascular density and foot blood flow associated with a rise in eNOS expression in the non-ischemic leg. By contrast, Senthilkumar et al. showed that sildenafil increases tissue blood flow only in the ischemic limb [20]. However, a major difference with this study was the use of a lower dose (10 mg/kg/d vs. 25 mg/kg/d in our study), in addition to species difference. We can assume that, at 25 mg/kg/d, sildenafil stimulates eNOS activity and NO may then activate PI3K-Akt signalling [29].

To confirm the effect of sildenafil on the non-ischemic hindlimb, we examined blood flow, structure, and protein expression in MRAs. Mesenteric blood flow, arterial diameter and eNOS activity increased in MRAs sildenafil-treated rats. As discussed above, sildenafil is a potent vasodilator when used chronically, increasing blood flow (and shear stress). Shear stress activates the PI3K-Akt pathway [35]. Akt phosphorylates eNOS [9], leading to increased NO production and subsequent endothelial cell growth and migration [10]. The chronic activation of the shear stress-dependent pathway induced arterial remodeling with a diameter enlargement, as shown in MRAs isolated from rats treated with sildenafil after both 7 and 21 days of treatment. These two models induced an increase in blood flow and artery remodeling. These findings support the hypothesis that sildenafil induces arterial remodeling in non-ischemic tissues. However, shear stress on endothelial cells in the vessel lumen is also highly likely to cause eNOS upregulation during ischemia-induced revascularization [36]. After ligation of the femoral artery, sildenafil also induced an increase in blood flow, vascular, and arteriolar density, but not in capillary density. This new results suggest that sildenafil induced a post-ischemic revascularization involving arteriogenis rather than angiogenis, as summerized in the figure below.

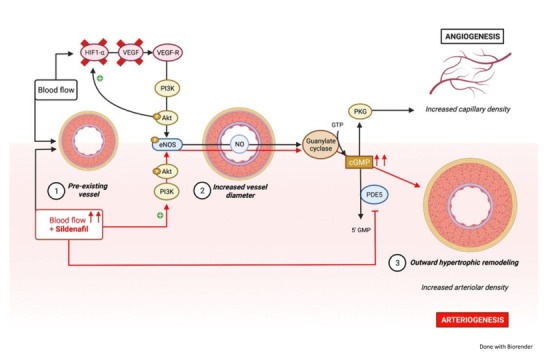



## 4. Materials and Methods

### 4.1. Animal Models

Animal studies were conducted according to the procedure followed in the care and euthanasia of animals and was in accordance with the European Community standards on the care and use of laboratory animals (authorization # 00577). Protocol was approved by the regional ethic committee of animal experimentation (authorization # 2008.10)

#### 4.1.1. Hindlimb Ischemia

Twelve-week-old male Wistar rats (Charles River, l’Arbresle, France) were housed in a regulated environment with a constant ambient temperature of 24 °C. They had free access to standard laboratory food and water. As previously described, the left femoral artery was ligated (3–0 silk suture) under anesthesia (sodium pentobarbital, 50 mg/kg, intraperitoneally [IP]) [37]; ligation was performed 0.5 cm proximal to the bifurcation to the saphenous and popliteal arteries. After 7 and 21 days of ligation, blood flow and vascular density were measured in anesthetized rats as described below, followed by euthanasia and tissue sampling. The results of revascularization are expressed as the ratio of left (L) (ischemic) to right (R) (non-ischemic) hindlimb (L/R ratio).

#### 4.1.2. Treatment

Animals were treated with a high dose of sildenafil (25 mg/kg by daily gavage), as described previously [38]. Control rats received 1 mL of water under the same conditions. To assess the involvement of VEGF, four additional groups were created: a control group that received 1 mL of water by daily gavage for 7 days; a control group treated with VEGF-neutralizing antibody (2.5µg, IP twice a week, R&D System); and two groups treated with sildenafil, with or without a VEGF-neutralizing antibody [39]. Treatment started 24 h after surgery and was continued for 7 or 21 days.

#### 4.1.3. Groups of Animals

For 7 days of experiments: four groups

1. CT, (control rats, N = 12)
2. CT + anti-VEGF (N = 12): control rats treated in vivo with anti-VEGF for 7 days.
3. Sildenafil (N = 12): control rats treated in vivo with sildenafil for 7 days.
4. Sildenafil + anti-VEGF (N = 12): control rats treated in vivo with sildenafil and anti-VEGF for 7 days.

For 21 days experiments: two groups

1 CT, (control rats, N = 12)
2 Sildenafil (N = 12): control rats treated in vivo with sildenafil for 21 days.

### 4.2. Blood Pressure and Mesenteric Blood Flow Measurements

After anesthesia with sodium pentobarbital (50 mg/kg, IP), the right carotid artery was cannulated to measure blood pressure [40]. A medial laparotomy was then performed. Body temperature was maintained at 37.5 °C by a thermostatically controlled heating platform. A section of the ileum was extracted and spread over a gauze swab that had been dampened with a sterile physiological salt solution (PSS). A segment of an MRA was dissected free of fat and connective tissue, and a transit-time ultrasonic flow probe (0.5 mm V series, Transonic Systems) was then placed around the artery. Following this, flow was measured over a period of 10 min (T106 flowmeter, Transonic Systems) as previously described [34].

### 4.3. Laser Doppler Flowmetry

Rats were settled in an incubator (MMS, Chelles, France) allowing a stable skin temperature to be maintained throughout the experiment. Foot perfusion was then measured using a laser Doppler flow probe (PF408, Periflux, Perimed, Sweden) with a 12.6 mm^2^ circular contact surface area, connected to a laser Doppler flowmeter (PF4001 Master, Periflux, Perimed, Järfälla, Sweden). With the probe held perpendicular to the skin, blood flow was recorded over 5 min. Two flow measurements were made per foot.

To evaluate the neovascularization at day 7, laser Doppler perfusion imaging experiments were performed as previously described [37]. Blood flow was calculated in the foot and expressed as a ratio of ischemic to non-ischemic leg (L/R ratio).

### 4.4. High-Definition Microangiography

Rats were anesthetized (sodium pentobarbital, 50 mg/kg, IP) and a contrast medium (barium sulfate, 6 g/mL) was injected at physiological pressure through a catheter connected to the abdominal aorta, as previously described [37]. Two images per animal were acquired using a digital X-ray transducer (Faxitron, Tucson, IL, USA). Vascular density was determined using Histolab (Microvision Instruments, Evry, France), and expressed as percentage of pixels per image occupied by vessels in a zone delineated by the ligature, the knee, the edge of the femur, and the external limit of the leg [37].

### 4.5. Histology

Microangiographic analysis was completed by assessment of capillary and arteriolar densities. Ischemic and non-ischemic muscles were dissected and progressively frozen in isopentane cooled in liquid nitrogen. Muscle sections of 7 µm [41] were incubated with mouse monoclonal antibody directed against human smooth muscle actin 1 (DakoCytomation, dilution 1:50) to identify arterioles, and with a rabbit polyclonal antibody directed against total PECAM-CD31 (Pharmingen, dilution 1:50) to identify capillaries. Capillary and arteriolar densities were calculated in five randomly chosen fields of a definite area for each animal.

### 4.6. Endothelial Cell Migration Assays

Boyden chamber assay was used to analyze the migration of human microvascular endothelial cells (HMEC, Lonza, Switzerland). HMEC were serum-starved overnight then trypsinized and washed in serum-free medium before plating into Transwell inserts (2 × 10^5^ cells per/insert, 8 μm pore size, Corning Incorporated Costar) in serum-free medium. Sildenafil (10 µM) [24] was added to the wells containing the Transwell inserts. To inhibit PI3K, cells were pre-incubated with 25 µM LY 294002 for 30 min. HMEC were allowed to migrate for 8 h at 37 °C. The filter was then removed, and the HMEC on the upper side of the filter were scraped off. HMEC that had migrated to the lower side of the filter were fixed in methanol at 4 °C for 15 min, stained with toluidine blue, and counted under a microscope. Migration was quantified by the number of migrated cells, expressed as a percentage of control.

### 4.7. Western Blot

MRA and skeletal muscles from both ischemic and non-ischemic legs of each rat were collected separately, frozen and then homogenized. Proteins were separated by SDS-PAGE. In the ischemic and non-ischemic muscle, eNOS, phospho-eNOS, caveolin-1, Akt, Phospho-Akt (P-Akt), PI3K, hypoxia-inducible factor 1 alpha (HIF-1α) and VEGF were then visualized with ECL-Plus Chemiluminescence kit (Amersham, United Kindom).

Antibodies directed against eNOS (1:500; BD 610297), anti-P-eNOSser1177 (1:500; BD 612392) and caveolin-1 (1:1000, BD 610407) were purchased from BD Transduction Laboratories (United States); Akt (1:1000, CellSignaling #9272), and P-Akt-ser473 (1:1000, CellSignaling #9271) antibodies were from Cell signaling (United States); and PI3K (p85α 1:500; sc-1637) and VEGF (1:500; sc-7269) antibodies from Santa Cruz (United States).

### 4.8. cGMP Concentrations

The concentrations of cGMP were measured as previously described [42] in plasma and aorta by Enzyme-Linked Immunosorbent Assay (ELISA) using a commercial kit (Cayman Chemical Co., Ann Arbor, MI, USA). Samples were initially precipitated with trichloroacetic acid, extracted with water-saturated ethyl ether and reconstituted in assay buffer.

### 4.9. Arterial Diameter Measurement in Isolated Arteries

After 21 days, MRA segments were cannulated at both ends in a video-monitored perfusion system (LSI, Burlington, VT, USA) [41]. Briefly, arteries were bathed and superfused with a Ca^2+^-free PSS containing EGTA (2 mmol/L) and sodium nitroprusside (10 µmol/L). Diameter changes were measured when intraluminal pressure was increased from 10 to 150 mm Hg.

### 4.10. Statistical Analysis

Results are expressed as mean ± SEM (standard error of mean). The significance of the differences between groups was determined by analysis of variance (ANOVA), following Bonferroni’s test and unpaired t-test. *p* values less than 0.05 were significant [37]. To analyze the endothelial cell migration assays, a paired t-test was performed.

## 5. Conclusions

In conclusion, this study showed for the first time that long term treatment with sildenafil appears to improve revascularization, activating the Akt-eNOS pathway in a VEGF-independent manner. We also showed that sildenafil induced the outward remodeling of resistance arteries. Thus, sildenafil might be an interesting therapeutic tool in occlusive peripheral arterial disease, and, more generally, in the treatment of diseases associated with vascular lumen narrowing, including hypoxia-induced pulmonary hypertension.

## Figures and Tables

**Table 1:** Body weight and blood pressure.

	Controls Rats (n = 10)		Sildenafil-Treated Rats (n = 10)	
	7 days	21 days	7 days	21 days
Body weight (g)	279 ± 8	426 ± 5	272 ± 6	418 ± 5
Mean arterial blood pressure (mmHg)	107 ± 3	115 ± 4	107 ± 2	100 ± 2 *

Legend: Data are given as the mean ± SEM. Mann–Whitney test was used. (* *p* < 0.05 compared to controls rats).

**Table 2:** Plasma and aortic cGMP contents.

	Controls Rats (*n* = 5)	Sildenafil-Treated Rats (*n* = 10)
Aorta cGMP (nmol/mg)	20.48 ± 0.88	63.96 ± 10.18 *
Plasma cGMP (nmol/mL)	17.12 ± 2.48	32.82 ± 4.69 *

Legend: Data are given as the mean ± SEM. Mann–Whitney test was used. (* *p* < 0.05 compared to controls rats). cGMP: guanosine 3′,5′-cyclic monophosphate.

## Data Availability

Not applicable.

## Abbreviations

(L)	Left
(R)	Right
Akt	Ak strain transforming
cGMP	Guanosine 3′,5′-cyclic monophosphate
CT	Control
EGTA	Ethylene glycol-bis(β-aminoethyl ether)-N,N,N′,N′-tetraacetic acid
ELISA	Enzyme-Linked Immunosorbent Assay
eNOS	Endothelial nitric oxide synthase
HIF-1	Hypoxia-Inducible Factor-1
HMECs	Human microvascular endothelial cells
IP	Intraperitoneal
MRA	Mesenteric resistance arteries
NO	Nitric oxide
PDE5A	Phosphodiesterase 5A
PECAM	Platelet endothelial cell adhesion molecule
PI3K	Phosphatidylinositol 3-kinase
PSS	Physiological salt solution
S	Sildenafil
SEM	Standard error of mean
TGF-β1	Transforming growth factor beta 1
VEGF	Vascular endothelial growth factor
